# Redefining tuberculosis: an interview with Lalita Ramakrishnan

**DOI:** 10.1242/dmm.050189

**Published:** 2023-03-23

**Authors:** Lalita Ramakrishnan

**Affiliations:** Molecular Immunity Unit, Cambridge Institute of Therapeutic Immunology and Infectious Diseases, University of Cambridge, Cambridge CB2 OQH, UK, and MRC Laboratory of Molecular Biology, Francis Crick Avenue, Cambridge CB2 OQH, UK

## Abstract

Professor Lalita Ramakrishnan is at the forefront of modern tuberculosis (TB) research. She has developed vital tools, most notably a robust zebrafish model, to study this disease, leading to seminal discoveries uncovering bacterial and host interactions throughout infection. Her group has harnessed this knowledge to develop new treatments for TB and shape clinical research. By unveiling these complex interactions, they have also improved our understanding of fundamental biology of macrophages and other infectious diseases, such as leprosy.


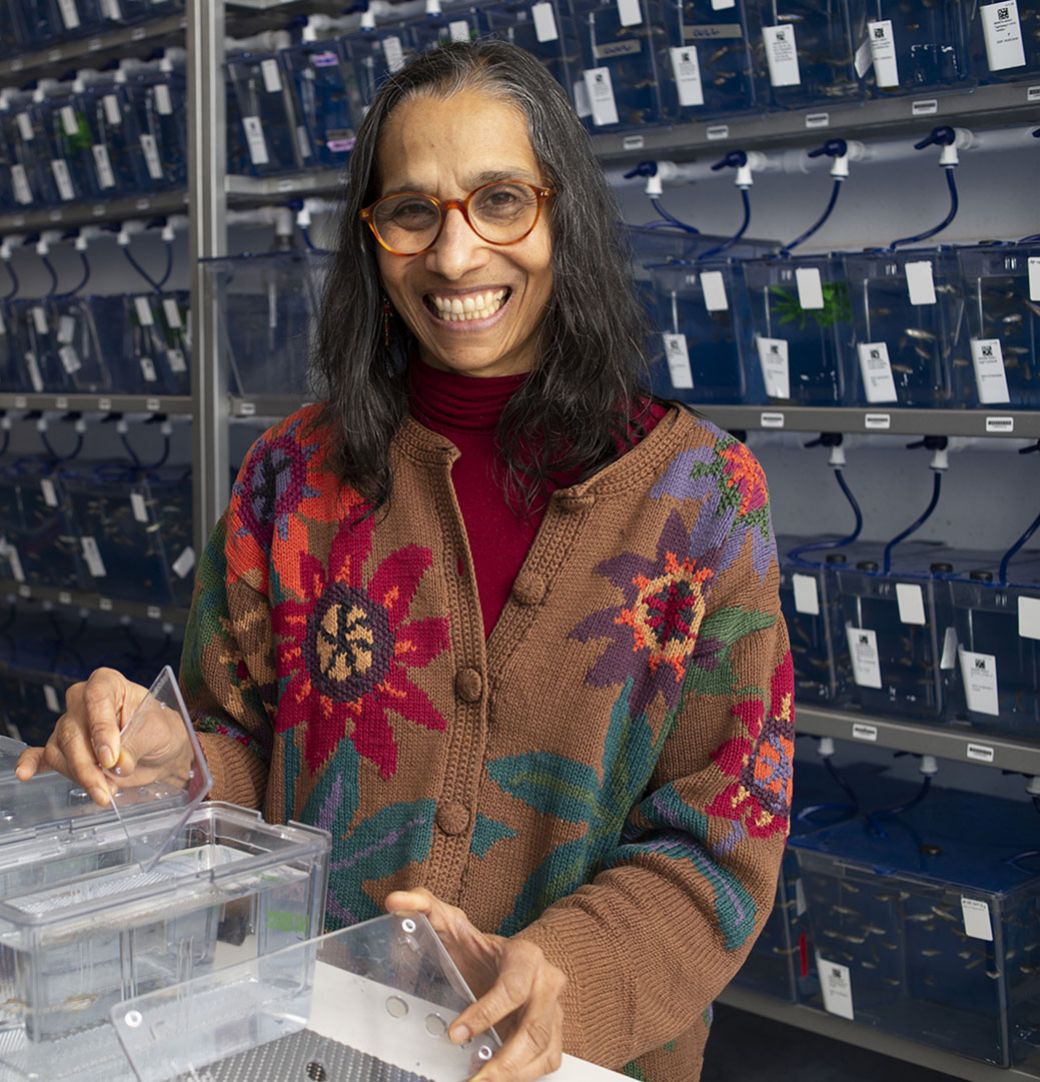


**Lalita Ramakrishnan.** Image courtesy of MRC Laboratory of Molecular Biology. This image is not published under the terms of the CC-BY license of this article. For permission to reproduce, please contact MRC LMB.

Early in her studies, Lalita oscillated between medicine and scientific research, building a holistic view of infectious disease. She is now based in the University of Cambridge and the MRC Laboratory of Molecular Biology (LMB), Cambridge, UK. She has received many awards and honours, including a National Institutes of Health (NIH) Director's Pioneer Award and a Wellcome Trust Principal Research Fellowship. She is a member of the US National Academy of Sciences and the European Molecular Biology Organisation, and a Fellow of the Royal Society, the Academy of Medical Sciences and the American Academy of Microbiology. In this interview, we discuss her career journey, the inspiration behind many of her key findings and her flexible approach to mentoring. As we acknowledge World TB Day, Lalita also highlights promising areas of research that will impact the future of TB prevention and therapeutics, focusing attention on the questions most relevant to TB in high-burden areas.

**Having trained and worked in several countries, how do you think these different medical and scientific environments have shaped your research today?**

Getting my initial medical degree in India, with the required year-long internship, taught me resilience and admiration for clinicians functioning under difficult circumstances, often with very limited resources.

Following on from this, training in the USA was very significant in my development as both a scientist and a clinician. In India, medicine is a 6-year undergraduate course. I then went to the USA and did a PhD in immunology at Tufts University. The USA PhD training programmes involve advanced level courses, which, for me with my patchy basic science background, were essential. Most important, of course, was my thesis advisor Naomi Rosenberg, from whom I learned to be a scientist. During my PhD, I realized I wanted to be a physician scientist and was fortunate to be accepted to the superb medicine residency program at Tufts-New England Medical Centre, where I was mentored by gifted and renowned physicians. I then did a clinical infectious diseases fellowship at the University of California San Francisco (UCSF), where I again interacted with stalwarts in infectious diseases. Then, my fellowship research was at Stanford University with Stanley Falkow, an absolute leader in bacterial pathogenesis, arguably my greatest scientific influence.

Starting my own lab at the University of Washington (UW), I was able to recruit amazing graduate students who were creative and fearless. Perhaps even more surprisingly, top-notch postdoctoral scientists joined my fledgling lab. I guess there were ambitious and adventurous scientists for whom the promise of a completely new model to tackle an important and difficult disease overrode the risk of working with a newbie principal investigator. It didn't hurt that UW had a great reputation with superb microbiology and immunology, and a cohort of excellent zebrafish researchers, all in one of the world's most beautiful cities. I was in the right place at the right time. Fortunately, my lucky streak with graduate students, postdocs and collaborators has followed me to Cambridge!

**What inspired you to develop the zebrafish model of TB, using *Mycobacterium marinum*, and why is it such a useful model of human TB?**

During my clinical fellowship at UCSF, I decided I wanted to study TB, an important infectious disease, the ravages of which I had seen up close in India, including in my mother, who had TB of the spine that relapsed twice. I also found TB biologically fascinating, as the TB bacterium has a complex lifestyle, much of which is living inside macrophages. I was aware of Stanley Falkow at Stanford, who was well known for his work on many different bacterial pathogens, several of which had intracellular lifestyles, just like the TB bacterium. So, even though he did not study TB, his lab seemed like a natural fit. But he initially dissuaded me, ‘We can't work with TB. I don't have the biosafety level 3 facilities here, and it's so slow growing that I'll be dead before you get your first result!’ He then suggested I look for a surrogate mycobacterium and told me of one that infects fish and frogs. Searching through Bergey's manual of systemic bacteriology, I saw he might have had *Mycobacterium marinum* in mind. Armed with a patient isolate from the UCSF clinical microbiology lab, I got started in his lab, figuring out how to work with mycobacteria, developing basic genetic tools in *M. marinum*, and understanding its behaviour in cultured macrophages. I developed an infection model in leopard frogs (*Rana pipiens*). These frogs are interesting because they develop chronic infection with characteristic granulomas of TB, but don't become sick and die unless you suppress their immune system with steroids. Unlike zebrafish, they're very much able to control the infection, which is something that still intrigues me.

Meanwhile, the idea to use the zebrafish had already been seeded by Don Payan, then on the UCSF Infectious Diseases faculty, with whom I had worked during my fellowship. I still remember this moment like it was yesterday. I was sitting with Don one evening, in his backyard, and he said, ‘So, what are you going to do next year for your research?’ I told him that I was planning to develop a model of TB using *M. marinum,* which infects cold-blooded animals. He said, ‘You could use zebrafish to explore the host side.’ I had no idea what he was talking about and quickly did some reading to realize that the zebrafish's genetic tractability made it an attractive model organism.

Towards the end of my postdoc, the stars were aligned to finally try Don's idea. Will Talbot, a zebrafish researcher who had just arrived at Stanford, suggested I try infecting the larvae, which are transparent. My lab bay mate, Nina Salama, had a zebrafish expert friend, Dori Hosobuchi, who had also just arrived at Stanford. We got larvae from Will and infected them with fluorescent *M. marinum*. Looking at the infected larvae under a microscope on a Saturday, Nina and I saw some cells whooshing around in the circulation that were infected with the bacteria. I did one more experiment before I left for the University of Washington. I was ready to start work in earnest on the zebrafish larval model, primed to benefit from the cohort of terrific zebrafish researchers there.

**You have translated many of your findings in zebrafish to human studies. How do you go about transitioning this research and what are the main challenges?**

First off, we centre our research on the mechanisms of disease pathogenesis and drug tolerance without seeking a ‘translational’ angle. Of course, even though we use the zebrafish as a guidepost, the relevance of the findings to human disease is very important to me. This can come in different flavours. Sometimes they explain a well-known but poorly understood feature of TB. For example, why does TB infection begin in the lower lung rather than in the upper airways as do many respiratory infections, including COVID-19? It turns out that virulent mycobacteria have two related lipids on their surface that collude to get the bacteria into their safe niche within the host. One staves off microbicidal macrophages that would kill them, whereas the other calls in permissive macrophages that ferry them across into deeper tissues where they can establish the infection focus. However, the upper airways have an abundance of resident bacterial flora that continually bring these microbicidal macrophages that the mycobacteria can avoid, which would of course kill the mycobacteria. So for the mycobacterium's dual lipid strategy to work, it needs to go straight down into the lungs, which are relatively sterile. Our lungless zebrafish larvae have provided a beautiful and satisfying explanation for this age-old puzzle (Cambier et al., 2014). Similarly, our foray into using the zebrafish to study leprosy provided insight into the mechanism of its terrible, disfiguring neuropathy (Madigan et al., 2017). The leprosy bacterium has a modified version of the macrophage-recruiting lipid on its surface, which does double duty. Like its TB counterpart, it recruits permissive macrophages. But it also causes dysregulated inflammation in infected macrophages, which then damage nerves as they are ‘patrolling’ them.

“Even though we use the zebrafish as a guidepost, the relevance of the findings to human disease is very important to me. This can come in different flavours. Sometimes they explain a well-known but poorly understood feature of TB.”

Then there were basic studies that led us to exciting clinical studies. We used the zebrafish model to do a forward genetic screen for host susceptibility pathways. Genetically induced overexpression of an enzyme in the arachidonic acid metabolism pathway called leukotriene 4 hydrolase (LTA4H) led to overproduction of a highly pro-inflammatory lipid called leukotriene B4, which caused hypersusceptibility to TB (Tobin et al., 2010, 2012). We then showed that a common human LTA4H variant dramatically impacted the survival probability of people suffering from TB meningitis, the most lethal form TB (Thuong et al., 2017; Whitworth et al., 2021a). Importantly, anti-inflammatory steroids were life-saving in people with this pro-inflammatory allele. Using zebrafish, we had identified an ideal example of genotype-guided host-targeting treatment for TB.

Then, using zebrafish, we have understood why overproduction of the pro-inflammatory lipid is bad, as it impacts the expression of tumour necrosis factor (TNF). This causes infected macrophages to die by a process called necrosis, which releases the bacteria into the extracellular milieu where they can grow very well, better than in macrophages (Roca et al., 2019, 2022). We identified a new cell death pathway, which involves cross-talk between multiple organelles such as the mitochondria, lysosomes and the endoplasmic reticulum in the infected macrophage, and the exact mechanism of this pathway. We also identified lots of drugs that intercept the pathway at different points, which are all available and safe to use for other conditions. One of these is metformin, used for the treatment of diabetes and, nowadays, by Silicon Valley techies hoping to live longer. In subsequent work, we have identified that excess TNF may be a larger problem. The excess TNF is probably caused by genetic variants in addition to the LTA4H-high variant we had identified. So drugs like metformin may benefit people with TB more broadly (Whitworth et al., 2021b).

A vexing aspect of TB is the need for months-long treatment with multiple antibiotics. This is because the TB bacteria become transiently ‘tolerant’ to the administered drugs even though they have not developed genetic resistance. We identified a mechanism for this tolerance, initially in zebrafish but rapidly moving our focus to human macrophages infected with the human TB bacterium. We found that upon infecting macrophages, mycobacteria turn on bacterial efflux pumps, which help them survive in these hostile cells, perhaps by pumping out noxious compounds (Adams et al., 2011; Lake et al., 2023). We then found that verapamil inhibits these bacterial efflux pumps, leading to reduced tolerance to the frontline TB drugs rifampicin, isoniazid and bedaquiline (Adams et al., 2014). Verapamil also inhibits the bacteria from growing in macrophages even in the absence of rifampicin. Bill Bishai and colleagues at Johns Hopkins University then showed that adjunctive verapamil could shorten treatment time in mice (Gupta et al., 2013). A phase 1 verapamil dose-finding study has recently been completed in India. We've also identified that proton pump inhibitors, used for acid reflux, are effective inhibitors of rifampicin efflux and, like verapamil, they inhibit tolerance and macrophage growth (Lake et al., 2023).

Verapamil is particularly interesting because it has two functions: in addition to blocking calcium channels, for which it is used clinically, it also blocks a eukaryotic drug efflux pump, P-glycoprotein (P-GP, or ABCB1). We've shown that verapamil inhibits bacterial efflux pumps through the same mechanism as it does P-GP (Lake et al., 2023). We also found that verapamil also inhibits the harmful macrophage cell death pathway by decreasing cellular calcium concentration (Roca et al., 2019). So verapamil could be beneficial in TB treatment by inhibiting bacterial drug tolerance through one mode of action and inhibiting macrophage death through another. This is an example of the power of basic research focused on understanding the disease. We did not set out to study verapamil!

“[…] if you're working on a disease in under-resourced parts of the world where the disease is common, it's even more difficult. If we had potential host-targeting treatments for a disease that is prevalent in well-resourced areas like the USA or the UK, I think it would still be slow, but progress would be faster.”

We've been blessed to work with superb, dedicated clinicians and clinical researchers in Vietnam, India and, more recently, Indonesia. For instance, the verapamil study was undertaken in India by Soumya Swaminathan and Padmapriyadarsini. Our pro-inflammatory lipid and TNF findings were recapitulated in patient cohorts in Vietnam and Indonesia (Whitworth et al., 2021a). Guy Thwaites, who heads the Oxford Wellcome Trust Unit in Vietnam, and his colleagues are doing a clinical trial to stratify the use of steroids in patients based on their *LTA4H* genotype (Donovan et al., 2018). Clinical work is slow, no matter where you are, but if you're working on a disease in under-resourced parts of the world where the disease is common, it's even more difficult. If we had potential host-targeting treatments for a disease that is prevalent in well-resourced areas like the USA or the UK, I think it would still be slow, but progress would be faster.

**You published two influential papers that debunked the misconception that *Mycobacterium tuberculosis* often persists in infected but asymptomatic individuals for many decades (‘latent TB’) and that a large proportion of the world's population has latent TB (Behr et al., 2018, 2019). Did you face much resistance to these discoveries from other researchers?**

The idea that a quarter or third of the world has latent TB was deeply entrenched in the collective consciousness. I used to teach this to medical students, and it is in the introduction of a couple of my early papers. But then I started to find many old studies that countered this dogma.

I got Marcel Behr and Paul Edelstein, both TB experts with a keen understanding of epidemiology, to join me in the quest to understand the natural history of TB. Digging into the literature, we found beautiful studies that showed that, in fact, most people self-clear TB, generally within weeks to months, going up to 2 years. However, they tend to retain an immunological stamp of TB infection that is manifested by a positive TB skin test, which has led to the erroneous conclusion that they are still infected. This understanding has profound scientific and public health ramifications.

There was some resistance to our analyses and conclusions, but overall, the response has been positive. We have each been invited to present the findings at key meetings. The World Health Organisation has changed what they say about latent TB in response to our work. They've published a corrigendum and their new guidelines include revised wording around latent TB. They are planning to do more, explaining the basis of the changes. So, I would say that overall, there has been substantial agreement with our analyses and recognition that they are reframing not just terminology but key questions in TB.

“If you think that a quarter of the world population – around 2 billion people – is infected with *M. tuberculosis*, it is a daunting challenge for eliminating TB. The realization that the true number, although still substantial, is much lower makes the problem more solvable.”

**Can you explain why these findings are important to redirect the focus in TB research and funding?**

If you think that a quarter of the world population – around 2 billion people – is infected with *M. tuberculosis*, it is a daunting challenge for eliminating TB. The realization that the true number, although still substantial, is much lower makes the problem more solvable. Also, the recognition that most TB cases manifest within 2 years has important practical implications. Prevention strategies should focus on people who have had recent exposures rather than those who have been harbouring a positive skin test for decades, who most likely are no longer infected. It can also guide vaccine trials, which can be daunting if you think you have to follow people for 20 years to see the benefit versus following people for a year or two.

From a research standpoint, we need to focus the questions on why the majority of people clear TB, but 5-10% of people don't. What is different about these people? There are many unknown risk factors, which, of course, is something that we are investigating with our genetic screens in zebrafish.

**You mentored many fantastic scientists, including Disease Models & Mechanisms Editor David Tobin. Do you have any strategies in mentorship?**

First off, I have gotten outstanding students and postdocs, and even technicians and undergraduate researchers who have often made key contributions. So, my job has generally been easy. I think that mentoring should be inherently flexible, tailored to the individual. I believe in light mentoring – benign neglect is how a lab member describes my mentoring style. At the same time, you want to be interested and engaged so that you can add value to the research, especially at key junctures.

I try to ingrain in my trainees a sense of what it means to be rigorous in science. Rigour should never be confused with being overly cautious, as that can become paralysing and thwart creativity.

“By building people’s confidence and encouraging intellectual freedom, you benefit too, because then everyone in the lab is contributing ideas. It's a win-win strategy. I count on the collective brain power of our group. It would be more than a little intimidating to run the show all by myself.”

Something I have observed is that often trainees just don't realize that they're terrific scientists, or that their thinking and ideas are valid and important. Women tend to suffer even more from this lack of confidence. So, it is important to imbue confidence, and this is best done by trying to elicit their ideas, being excited about their good ideas, and reminding them of their successful ideas. For many of us, confidence comes slowly and is built on past success.

By building people’s confidence and encouraging intellectual freedom, you benefit too, because then everyone in the lab is contributing ideas. It's a win-win strategy. I count on the collective brain power of our group. It would be more than a little intimidating to run the show all by myself.

**What do you enjoy doing outside of the lab and work?**

I bicycle with a club, which is great fun and has made for friendships outside of work. I run and, when I can, hike and cross-country ski. I enjoy cooking, which helps keep up the pretence of ‘being good in the lab’. I enjoy doing these things, or just hanging out, with family and friends. I've lived in many different places and have maintained close friendships from each place and phase – starting from childhood and including former lab members. I have a global buddy network.
